# Yolo-Pest: An Insect Pest Object Detection Algorithm via CAC3 Module

**DOI:** 10.3390/s23063221

**Published:** 2023-03-17

**Authors:** Qiuchi Xiang, Xiaoning Huang, Zhouxu Huang, Xingming Chen, Jintao Cheng, Xiaoyu Tang

**Affiliations:** 1 School of Data Science and Engineering, Xingzhi College, South China Normal University, Shanwei 516600, China; 20218131007@m.scnu.edu.cn (Q.X.); 20218131003@m.scnu.edu.cn (X.H.); 2 Department of Computer Science, Aberystwyth University, Aberystwyth SY23 3FL, UK; zhh6@aber.ac.uk; 3 School of Electronics and Information Engineering, South China Normal University, Foshan 528000, China; 2021022364@m.scnu.edu.cn; 4 School of Physics and Telecommunication Engineering, South China Normal University, Guangzhou 510006, China; 20172332035@m.scnu.edu.cn

**Keywords:** Yolo-Pest, data augmentation, small object detection, pest detection, lightweight, controllable channel

## Abstract

Insect pests have always been one of the main hazards affecting crop yield and quality in traditional agriculture. An accurate and timely pest detection algorithm is essential for effective pest control; however, the existing approach suffers from a sharp performance drop when it comes to the pest detection task due to the lack of learning samples and models for small pest detection. In this paper, we explore and study the improvement methods of convolutional neural network (CNN) models on the Teddy Cup pest dataset and further propose a lightweight and effective agricultural pest detection method for small target pests, named Yolo-Pest, for the pest detection task in agriculture. Specifically, we tackle the problem of feature extraction in small sample learning with the proposed CAC3 module, which is built in a stacking residual structure based on the standard BottleNeck module. By applying a ConvNext module based on the vision transformer (ViT), the proposed method achieves effective feature extraction while keeping a lightweight network. Comparative experiments prove the effectiveness of our approach. Our proposal achieves 91.9% mAP0.5 on the Teddy Cup pest dataset, which outperforms the Yolov5s model by nearly 8% in mAP0.5. It also achieves great performance on public datasets, such as IP102, with a great reduction in the number of parameters.

## 1. Introduction

Insect pests are a significant threat to the yield and quality of crops. Accurate and timely detection of pests is crucial for the effective control of agricultural production. Currently, pest identification relies heavily on manual inspection, which is time-consuming, labor-intensive, and susceptible to error [1]. In natural field conditions, pest images face challenges such as inter-species similarity, varying scales, posture changes, illumination effects, and crop occlusion [2]. Traditional pest image detection and recognition methods have low accuracy and weak model generalization capabilities. In contrast, deep learning technologies and methods that have emerged in recent years have been widely used in the field of computer vision, including pest image detection and classification [3,4,5,6]. These methods have demonstrated strong performance in large-scale and fine-grained detection and recognition tasks and have become the preferred agricultural pest image detection solution. Therefore, exploring new methods for practical image detection tasks of crop pests and proposing more effective pest object detection algorithms is of great significance.

Existing convolutional architectures have achieved significant progress in computer vision tasks for various industrial scenarios, including object detection [7]. However, pest detection goes a step further than regular object detection due to the size, natural habits, and environment of the subject of the detection, which brings intractable challenges to current networks. Firstly, the limited availability of features presents a challenge in the feature extraction process for small objects. As the number of convolutional neural network (CNN) layers increases, the target feature information tends to become attenuated, making it difficult to obtain discriminative features. Additionally, the use of a multi-layer network may result in missed detections of some objects due to the weakening of target feature information. Secondly, the tendency of small objects to cluster together can pose challenges for object detection. This can occur due to the habits of pests, which often congregate under the capture device. Specifically, the small objects in the aggregation region may become aggregated into a single pixel on the feature map, making it difficult for the detection model to distinguish individual objects. Additionally, if the small objects in the aggregation region are too close together, it can be challenging to accurately regress bounding boxes, and the model may struggle to converge. Finally, the complex recognition background can present challenges for pest identification. This is often the case when the data used for identification is collected and processed by pest control devices, as the images may contain a variety of insects, incomplete pest corpses, and other background interferences. These factors can lead to issues such as missed detections and misdetections, which can result in a low recall rate and other problems during the detection stage. In particular, this problem further increases the negative impact brought by the lack of data. It is more difficult for the model to learn the characteristics of the corresponding pests in the face of very small amounts of data and low-quality data on certain pests.

To tackle the aforementioned problems, we propose our Yolo-Pest. Yolo-Pest is an improved one-stage object detector based on small target pest detection, which can effectively solve the problem of feature extraction of small target objects lost in the old detector. At the same time, both small-size and normal-size pests exist in the Teddy Cup pest dataset. Yolo-Pest can reduce the complexity of the model while reducing the loss of characteristics of small target pests and does not affect the detection of normal-size targets. There are many reasons why it is difficult to identify small target pests. For example, the depth of the model is too shallow to extract the features of a small target. The structure suitable for normal-size targets is easy to over-convolve and lose features when detecting small targets. The characteristics of small target pests are few, and it is difficult to extract valuable characteristics with a small amount of data. In this paper, we comprehensively considered various factors, enriched the characteristics of small target pests through data enhancement, replaced the feature extraction module in the model, reduced the excess framework for pest detection, and reduced the computational amount and time complexity of the model. The main contributions of this paper are:

- This paper, in the Teddy Cup pest dataset, explores a data enhancement method based on a complex background. Random background enhancement is used, which improves the robustness, generalization ability, and sensitivity to the interference of the model to a certain extent;
- This paper proposes the Yolo-Pest model for pest identification of various sizes and connects the ConvNext block [8] and SE [9] attention mechanism into the model. Large kernel sizes and other tricks are used to increase the receptive field to reduce the influence of the complex background and improve the overall model accuracy;
- This paper proposes a new module with the controllable channel residual feature extraction structure CAC3 (Convolution attention c3 module), which controls the stacking degree of residual blocks to solve the problem of feature loss or redundancy, explores the common improvement of object detection accuracy of different sizes in the model, and achieves an excellent balance between the accuracy of the model and the inference speed.

## 2. Related Work

### 2.1. Object Detection

Recently, much research has been conducted on pest detection and recognition based on deep learning technology. A convolutional neural network (CNN) is most commonly used for object detection tasks. A CNN-based detection approach is usually composed of three parts: the backbone network, the neck, and the detection head. Their backbone could be VGG [10], ResNet [11], SqueezeNet [12], or ShufflNet [13,14]. The above models are classical feature extraction models with suitable network depths and guaranteed performance. As for the head part, it is usually categorized into two types, i.e., a one-stage object detector or a two-stage object detector. The most representative two-stage object detector is the R-CNN series [15], which includes fast R-CNN [16], faster R-CNN [17], R-FCN [18], and Libra R-CNN [19]. These are extremely accurate but bulky models that are difficult to use in practice. As for one-stage object detectors, the most representative models are the YOLO series [20,21,22,23], SSD [24], and RetinaNet [25]. Most of the models used for implementation are one-stage detection models because they have the characteristics of fast speed, low computational amount, and low hardware requirements. This paper mainly explores the one-stage object detector.

The one-stage object detection algorithm is employed in various fields due to its performance in time and accuracy. The authors of [24] introduce the output space of bounding boxes into a series of origin boxes over different aspect ratios and scales. Additionally, the authors of [25] present a focal loss algorithm to solve the unbalanced screening of positive and negative samples. To improve the efficacy of one-stage object detection, Redmon et al. [21] proposed a novel approach utilizing a new backbone to accomplish one-stage tasks. This method is based on the Yolo model and its enhanced version, which utilize downsampling components, splicing components, or path aggregation networks to complete feature extractions of images with varying dimensions. This is achieved by directly mapping features of different sizes to target probabilities, boundary box locations, and object classification probabilities. The backbone is responsible for extracting features from the input; the neck is tasked with allocating and merging features to the head, which then detects objects using materials from the neck. Additionally, attention mechanisms, such as SPP [26], SE, CBAM [27], and CA [28], can enhance the efficiency and performance of detectors, especially for lightweight detectors. Yolov5s is a highly advanced algorithm developed from continuous improvement to the original model, which achieves the optimal balance between detection speed and accuracy and is suitable for embedding in actual machinery and equipment. However, as Yolov5s is only designed for public datasets, its feature extraction network is more suited for normal targets. The Teddy Cup pest dataset presents challenges, such as varying target sizes, complex background noise interference, and unbalanced sample classification. Therefore, this paper proposes an improvement to the backbone feature extraction network and the strengthened feature extraction network of Yolov5s and introduces the CAC3 block to address these issues.

### 2.2. Pest-Based Object Detection

The development of pest detection technology is related to crop loss investigation [29]. Wen et al. [30] presented a model designed for detecting pests. This study introduces three distinct methodologies: the first solely utilizes local insect image features, the second employs only global insect image features, and the third methodology leverages a combination of local and global features. The proposed model represents a straightforward yet effective approach to pest detection, with potential applications in agricultural settings and beyond. Siti N. A. Hassan et al. [31] presented a novel model for detecting two specific species of insects, namely Grasshoppers and Rhopalocera, by employing digital image analysis techniques. The model utilized a combination of the color and shape attributes of the insects as features for identification. To extract the shape of the insects, the RGB color space was first transformed into a binary format. While the effectiveness of this approach was demonstrated for the two targeted species, its applicability to a broader range of insect species remains to be evaluated. Xiaowen Huang et al. [32] introduced a new metric called truncated structurally aware distance (TSD), which is both simple and powerful. They also developed a truncated structurally aware distance loss function, which takes into account the distinct geometric relationships between two bounding boxes and utilizes a truncated function to impose varying penalties. This was proposed in recent times and has proven to be an effective approach.

## 3. Materials and Methods

In this section, a comprehensive account of our proposed methodology for developing a streamlined and effective model for pest detection will be presented. Our approach takes into account a range of factors, including the selection of convolutional methods, the development of feature fusion structures, computational efficiency, and cost-effectiveness, among other considerations, with the aim of maximizing the performance of the pest identification system.

### 3.1. Yolo-Pest Structure

The proposed solution in this paper, Yolo-Pest, represents an improvement to the Yolov5s model, which is a continuously improved and refined version of the YOLO series of target detection algorithms. Due to its high detection accuracy and fast detection speed for target objects, Yolov5s has been widely adopted in various industries. Yolov5s incorporates some minor innovations to the details of Yolov3, as described in Redmon et al. [22]. These improvements include: firstly, enhancing the feature extraction network through the use of the spatial pyramid pooling feature (SPPF) and the path aggregation network (PANet) instead of the feature pyramid network (FPN); secondly, replacing the Darknet53 backbone feature extraction network with CSPDarknet53 and the use of the SiLU activation function instead of LeakyReLU; and, finally, replacing the intersection over union (IOU) loss function for bounding box regression with the complete intersection over union (CIOU) loss function.

**Figure 1:**
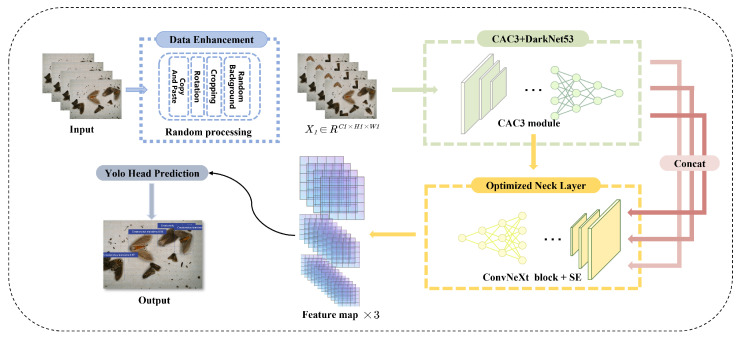
The structure of Yolo-Pest.

The Yolov5s model is selected for improvement because it is still being updated, and, after exploration in recent years, it has achieved remarkable results in various fields. It has the characteristics of being lightweight and having a fast detection speed. We hope to have an embedded model that can take into account the amount of calculation and accuracy and solve various problems in the actual operation of pest identification. To improve and explore the problems encountered in the detection of pest datasets, such as difficult convergence, feature redundancy, and background interference, we propose the Yolo-Pest model. The network structure diagram is shown in Figure 1.

The Yolo-Pest network is comprised three distinct components: the backbone feature extraction network, the enhanced feature extraction network, and feature prediction. The backbone feature extraction network utilizes the CAC3 module, an improved version of the residual module in CSPDarknet53, to iteratively reduce the size of the input image and extract more feature information from the image. The enhanced feature extraction network is comprised the spatial pyramid pooling feature (SPPF) and an optimized neck layer. The SPPF helps to increase the receptive field of the network and isolate the most relevant context features. The optimized neck layer utilizes the process of upsampling and downsampling to repeatedly extract features and enhance shallow features through feature fusion with the pyramid attention network (PANet). The optimized neck layer also employs the ConvNext block and SE attention mechanism to boost the representational power of the network. In the feature prediction part, the network outputs three enhanced features, and the loss function is calculated using both the actual data and the predicted bounding box, target category, and confidence value.

In this section, we provide a trajectory going from Yolov5s to Yolo-Pest. We consider two models in terms of FLOPs; one is the Yolov5x with FLOPs around 205.2 G, and the other is Yolov5s with FLOPs around 16.2 G. For simplicity, we will present the results with the Yolov5s complexity models. The conclusions for higher capacity models are consistent.

Our starting point will be the Yolov5s backbone: CSPDarkNet53. This network structure applies CSPNet [33] to DarkNet architecture, which outperforms the CSPResNext50 architecture on the COCO dataset. CSPDarkNet53 in Yolov5s consists of normal convolution and a C3 block. The Bottleneck module in C3 uses a convolution kernel of the size 1 × 1 to simplify the calculation and easily change the number of channels of the feature map. The experimental records show that the accuracy of the model in identifying small-size pests is much less than that of normal-size pests. We reasonably speculate that the stacking of Bottleneck modules in the backbone feature extraction network does not help to extract the feature information of small target pests. In order to verify this conjecture, we modify the number of channels in the input Bottleneck module of the C3 module, reduce the proportion of the Bottleneck output channels in a concatenate operation, and prove that the stacking of bottlenecks cannot play an effective role in small target pests. Based on the premise of reducing the computational complexity and simplifying the model, we modify the C3 module and replace it with the CAC3 (Conv Attention C3) module.

The PANet path-aggregation neck is used as the method of parameter aggregation from different backbone levels for different detector levels. To ensure the balance between feature extraction ability and network complexity, we explore the application of the ConvNext block in the model. Its architecture, mimicking the ViT (vision transformer) achieves 87.8% ImageNet top-1 accuracy and outperforms the Swin transformer on COCO detection and ADE20K segmentation while maintaining the simplicity and efficiency of standard ConvNets. The optimized neck layer with a replaced ConvNext block achieves results with reduced parameters and guaranteed high accuracy. Finally, we insert the SE attention mechanism module at the end of the output of the minimum feature map for normal-size object detection to achieve better results.

### 3.2. Data Augmentation

**Figure 2:**
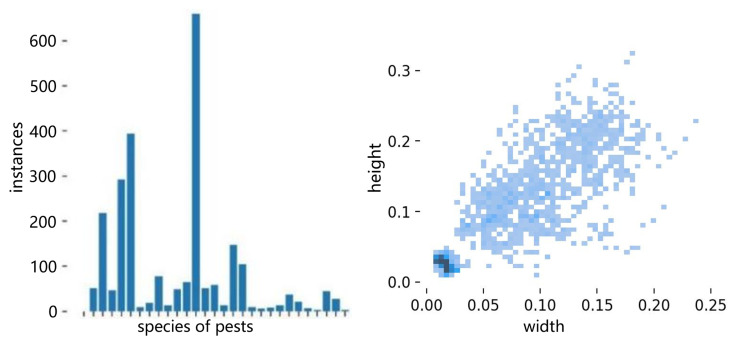
Statistics of the number of 29 pest labels and the length and width of all pest detection boxes.

The data in this paper comes from the 10th Teddy Cup National Data Mining Challenge for College Students (China) (https://www.tipdm.org/, accessed on 16 April 2022). The dataset consists of 576 images showcasing 28 different types of pests under probe light conditions. The sample data for pests exhibit a pronounced class imbalance issue, as depicted in the accompanying Figure 2. Of 1019 tags, Creatonotus transients had the highest number of samples, constituting 24.24% of the total data, followed by Nilaparvata at 14.72%. Conversely, Maruca textualism Geyer and Staurophora celosia Linnaeus had the lowest number of samples, only 0.09%. The sample imbalance presents a risk of the model overfitting to the classes with larger samples and disregarding the learning of smaller samples. Hence, it is imperative to employ suitable data augmentation strategies to increase the size of the dataset.

To enhance the model’s generalization capacity and robustness, data augmentation was performed by adding noise, flipping, and translation to the images while preserving the data’s characteristics. Additionally, we employed the copy-and-paste method [34]. This method involves balancing the categories of the targets that are to be cut out, selecting a random set of background images, and randomly pasting the clipped targets on the background images to address the issue of small sample numbers in the tail categories. The proportion of the target image in the background is increased, and the proportion of the original background is reduced, thus effectively resolving the label imbalance problem. This approach trains the network to learn the features of the target-background combination, thereby enabling better distinction between the foreground and background.

The dataset in this paper is collected by automatic insect trapping, insect killing, uniform arrangement, and photo taking of the insect situation searchlight. The quality of the collected pest data is easily affected by external reasons, such as the placement position and working time of the insect situation searchlight. The experimental data show that there are special insect conditions and complex backgrounds in some data, and it is difficult for the model to distinguish between pests and the background during training. This greatly affects the model’s learning of this part of the data features in recognition and affects the overall deep convergence and generalization ability of the model.

To guarantee the model’s recognition accuracy in complex backgrounds, we propose a data enhancement technique based on the complex background of the dataset itself: random background enhancement.

**Figure 3:**
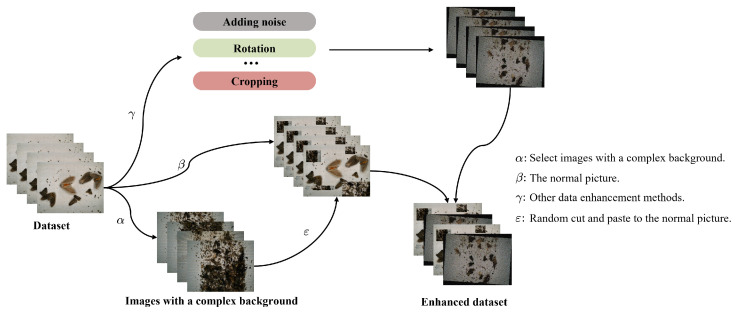
Flow chart of data enhancement in this paper.

As depicted in the accompanying Figure 3, a batch of noisy and interference-rich data is first selected, and images are randomly chosen from it. The background of the selected data is randomly cut and then rotated or scaled and pasted onto the remaining preprocessed images, covering the original background, while avoiding covering the target and excessive scaling. The pest dataset in this study was captured by taking photos from above the pests, thereby avoiding any confusion in a three-dimensional space due to random background enhancement. Most of the background interference is from insect residue and metabolites; thus, the selected background interference is applicable to most data, and there are no unrealistic data after enhancement. The enhanced dataset helps the model extract global features, increases the difficulty of model training, and forces the model to distinguish between the background and target, thereby improving its generalization ability. Overall, the random background augmentation in this paper is designed for all pest data collected using insect searchlights. However, this method can also be used for datasets that meet the following conditions:

- Some data in the dataset have complex interference and a lot of noise;
- Interference is reasonable for most of the data and may be present in all of the data;
- There is no spatial irrationality in changing the background position randomly or in a certain dimension.

After applying data augmentation to balance the sample categories and augmenting a large amount of data, we have 3198 images, which are divided into training, validation, and test sets in an 8:1:1 ratio.

### 3.3. CAC3 Module

In the experiments, it was found that the stacking efficiency of residual blocks is not high for feature extraction of small target pests in lightweight shallow networks. The addition of residual edges will lead to additional computational cost and time complexity. Therefore, based on the premise of reducing the amount of calculation and improving efficiency, we propose a controllable residual structure stacking module, the CAC3(Conv Attention C3) module.

The CAC3 module is based on the controllability of Bottleneck stacking. In order to improve computational efficiency in CNNs, a common preprocessing step is to transform the input images in such a way that the spatial information is gradually transferred to the channels. This process, known as spatial-to-channel transformation, typically involves a series of operations that compress the width and height of the feature maps and expand the number of channels. However, it is important to note that this process can result in a partial loss of semantic information as a consequence of the compression and expansion.

**Figure 4:**
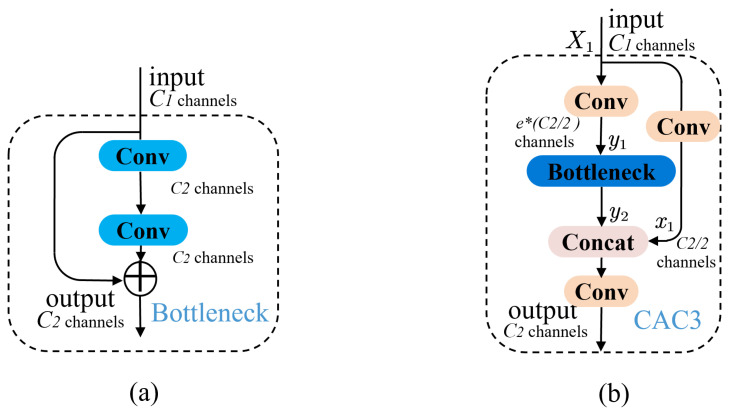
The structures of (**a**) Bottleneck and (**b**) CAC3 modules.

The Bottleneck appears for the first time in Resnet, and the structure is shown in Figure 4a. The Bottleneck greatly preserves the features in different channels with less computational cost and alleviates the problem of gradient disappearance caused by the increasing depth in deep neural networks. However, the target is small, and the extracted features are limited on the insect dataset. Stacking the Bottleneck structure does not work as well as it should.

As shown in Figure 4b, the feature maps extracted after the Conv block are fed into the CAC3 block. The feature maps $X_{1}\in R^{C1\times H1\times W1}$ are obtained after a 3×3 convolution. $C_{1}$ and $C_{2}$ are the numbers of input and output channels of the module. The module lets $X_{1}$ pass through the Conv block twice with different numbers of output channels to output two feature maps as $x_{1}$ and $y_{1}$. The spatial size of each feature map subset is the same as the input feature maps $X_{1}$, and the number of channels is $C_{2}/2$ and $e\cdot \left(C_{2}/2\right)$. As shown in Figure 4b, $y_{1}$ will be used as the input of the Bottleneck, and it will go through 1×1 and 3×3 Conv blocks, in turn, without changing the number of channels or the spatial size of the feature map. In the Conv block, it first convolves the input feature map; then, batch normalization is applied to it, and, finally, the output is normalized with the SiLU function.

(1) $$f_{conv}\left(x_{i},c\right)=\sigma \left(\rho \left(C\left(x_{i},c\right)\right)\right)$$

where *c* denotes the output channels; σ denotes the SiLU function; ρ denotes the batch normalization, which can accelerate the convergence speed of the network; and *C* denotes the convolution operation. The output result of two consecutive Conv blocks will be added to $y_{1}$ to produce the output $y_{2}$.

(2) $$g_{bottleneck}\left(y_{i},e\right)=y_{i}⊕f\left(f\left(y_{i},e\cdot \frac{C_{2}}{2}\right),e\cdot \frac{C_{2}}{2}\right)$$

where *g* denotes the output of the Bottleneck, and $y_{1}$ is added as a residual edge in the module with its own output feature map passing through two Conv blocks. We add a weight *e* to control the number of feature map channels passing through the Bottleneck structure.

(3) $$F_{CAC3}=f\left(concat\left(f\left(X_{i},\frac{C_{2}}{2}\right),g\left(X_{i},e\right)\right),C_{2}\right)$$

where *F* denotes the output of the whole CAC3. The weights in the CAC3 module can well balance the feature extraction ability of different size targets, the degree of feature loss, and the time complexity of the model. At the same time, in the face of small target pests, more features of each size feature layer in the network can be retained, and the quality of the input feature map of the neck part can be guaranteed. When the weight is reduced and the channel is changed, the parameters and FLOPs of the network model can be reduced.

### 3.4. Optimized Neck Layer

We find that the backbone after adopting the CAC3 module retains more feature information of small target pests, and the feature information extraction ability of the original neck part does not match it, so it cannot play a good role in enhancing the extraction function. Therefore, we add the ConvNeXt block in the neck layer.

**Figure 5:**
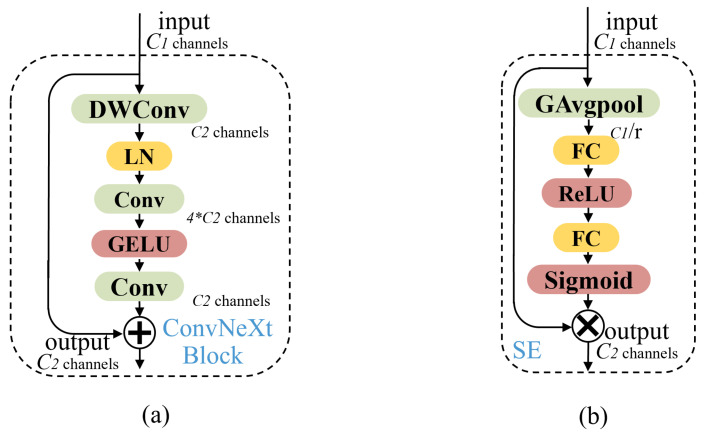
The structure of (**a**) ConvNeXt block and (**b**) SE attention mechanism.

The ConvNeXt block is shown in Figure 5b. Constructed entirely from standard ConvNet modules, ConvNeXt competes favorably with transformers in terms of accuracy and scalability. It “modernizes” a standard ResNet toward the design of a vision transformer and discovers several key components that contribute to the performance difference along the way. First, ConvNeXt explores why transformers perform so well and simulates and improves them with pure convolutional neural networks. In the Swin transformer, a convolution layer with a convolution kernel size of 4 × 4 and a stride of 4 is used to form a patchify. In order to reduce FLOPs and improve performance, ConvNeXt uses the patchify to replace the downsampling module stem. ConvNeXt then adopts the ResNeXt guiding idea of grouped convolutions in order to have a better FLOPs/accuracy trade-off. ConvNext uses deep convolutions, which are grouped convolutions with the number of groups equal to the number of channels, in which the convolutional filters are split into different groups, thereby reducing FLOPs and expanding the network width to compensate for the capacity loss. An important design of each transformer block is that it creates an inverted bottleneck, the MLP module. The MLP module is very similar to the inverted Bottleneck module in MobileNetV2 [35], so the inverted Bottleneck module is added to the ConvNeXt network. In transformers, it is common to perform global self-attention, such as with the vision transformer [36]. The Swin transformer has 7 × 7 windows, but the mainstream convolutional neural networks now use 3 × 3 windows. Based on this, ConvNext adopts two improvements, moving up depth-wise conv layer and Increasing the kernel size and then replacing it with a 7 × 7 convolution kernel to expand the receptive field, reduce the model’s FLOPs, and alleviate the problem of feature loss. Finally, in order to continue to simplify the model, ConvNext reduces the normalization. It replaces BN (batch normalization) with LN (layer normalization), which reduces the negative impact of BN layer stacking. The activation function ReLU, commonly used in convolutional neural networks, is replaced by the activation function GELU in the transformer, and the frequency of use is reduced. Finally, the Swin transformer implements downsampling by a single-patch merging, and ConvNeXt imitates it by designing a single downsampling layer, which is constructed by a layer normalization plus a convolution layer with a convolution kernel size of 2 and a step distance of 2.

The structure of the squeeze-and-excitation (SE) attention mechanism is shown in Figure 5a. The SE is a channel attention module, including two operational processes: the squeeze and the excitation. This module allows the networks to focus more on the more informative features’ channels and negates the less informative features’ channels. After increasing the detection accuracy of small target pests, in order to ensure the detection accuracy of normal-size pests, this paper inserts the SE attention module in front of the P2 detection head used to detect normal-size pests. This is due to the lack of low-level semantic information in the deep layer network, which can better use the information fusion function of the attention module.

## 4. Experiments

In this section, we conduct a detailed experimental analysis and verify the designed model on two different datasets, namely IP102 [37], and our own pest dataset which comes from the tenth Teddy Cup National Data Mining Challenge for College Students (China) (https://www.tipdm.org/, accessed on 16 April 2022). Additionally, the effectiveness of this method is demonstrated through experiments.

### 4.1. Datasets

- IP102

The IP102 dataset is a large-scale dataset for insect pest recognition. The dataset contains more than 75,000 images belonging to 102 categories, which exhibit a natural long-tailed distribution. It has 19,000 images with boxes with boundaries for object detection. It contains labels for categories such as rice leaf caterpillar, paddy stem maggot, and Cicadellidae.

- The Teddy Cup pest dataset

This dataset is a small-scale dataset for pest identification. The dataset contains 28 pest categories and a total of 3198 images. The background is an all-white background of insect pests collected by insect searchlights, and the number of insect pests varies greatly in the images. The size difference between species was also large. It contains pests such as Creatonotos transiens, helicoverpa armigeram, and Athetis epigone.

### 4.2. Experimental Details Settings

The configuration of the experiment is as follows: Intel Core i9-11900K, Ubuntu20.04 system, and NVIDIA RTX3090 graphics card using the CUDA framework 12.0 and the Pytorch 1.13 learning framework.

In the experiment, we adjusted the training parameters on the Yolov5s model to make the overall accuracy of the model reach a level that is difficult to improve; the same hyperparameters are used for the ablation experiment. Using the SGD optimizer, the weight attenuation coefficient is $5\times 10^{-4}$, the momentum is set to 0.937, and the initial learning rate is set to 0.01. The number of iterations on all datasets is 300. The learning rate is attenuated using the cosine annealing algorithm.

(4) $$new\_lr\;\;=eta\_min+\left(initial\_lr-eta\_min\right)*\left(\left(1+\cos\left(\frac{cur\_epoch}{T\_max}*\pi \right)\right)/2\right)$$

where eta_min denotes the minimum learning rate; initiail_lr denotes the initial learning rate; and cur_epoch and T_max denote the current epoch and the total number of epochs, respectively. With the increase in the number of iterations, the learning rate shows a trend of first increasing and then decreasing. To avoid overfitting, for IP102 and the Teddy Cup pest dataset, we used the methods of adding noise, flipping, panning, random cropping, copy-and-paste, and random background enhancement mentioned in Section 3.2 of this paper for data enhancement.

### 4.3. Ablation Experiment

In Table 1, we test the accuracy of DarkNet53 built with CAC3 modules, and the backbone built with different modules in the Teddy Cup pest dataset (in order to be fair, we use the same hyperparameters). These methods first include inserting the C3 and Hornet blocks [38] into DarkNet53, respectively. Then, Mobilenet [39] and Regnet [40] are used as the backbone of the model for comparison. We conducted comparative experiments in the CAC3 model when the weight *e* was 0.25 and 1.0. When *e* is 0.25 and 1.0, we can control the channel ratio of the output residual edge and residual structure in the feature map concatenated as 2:1 and 1:2, respectively. In the Teddy Cup pest dataset, the CAC3 algorithm obtains the best result with low time complexity when *e* is 0.25. The experimental results show that when *e* is 1.0, under the premise of not processing residual edges and preserving the features as much as possible, increasing the number of feature channels through the residual structure cannot improve the accuracy of datasets with small targets and will increase the number of model parameters and the amount of calculation. Adjusting the convolutional output channels to reduce the number of channels passing through the residual structure not only can reduce the side effects caused by the stacking of residual structures but can also reduce the operation cost and model complexity.

Further, in Table 2, we verified the accuracy of the ConvNeXt blocks inserted into the enhanced feature extraction network in the Yolov5s network and compared them with other feature extraction modules. The methods for comparison include a ConvNeXt block and a Hornet block, which replace a Bottleneck module and a C3 module in the model, respectively. The results show that the best performance occurs when the ConvNeXt block replaces a Bottleneck in the C3 structure. Compared to the method of replacing a C3 block directly with a Hornet block, the number of parameters is significantly reduced, and the average accuracy is higher. The ConvNeXt block reduced computational costs and achieved good model performance improvements. In Table 3, we report the experimental results of Yolov5s using different attention modules. It is found that, except for the SK attention modules, different attention modules have little influence on the number of detector parameters and inference time, which can almost be ignored, but their influence on the accuracy is significant. The SE attention mechanism achieved the best results for the number of parameters in the Teddy Cup pest dataset, noting that we only added an attention module before the feature graph output for normal-size object detection.

To prove the effectiveness of the proposed method after fusion, the relevant results are shown in Table 4. We use Yolov5s for a baseline intuitive comparison. After adding each module, the accuracy of the model continues to improve. We set a comparative experiment for the weights of different channels and found that when tested on the Teddy Cup pest dataset, e = 2.75, and the model accuracy and computation amount achieved a good balance.

**Figure 6:**
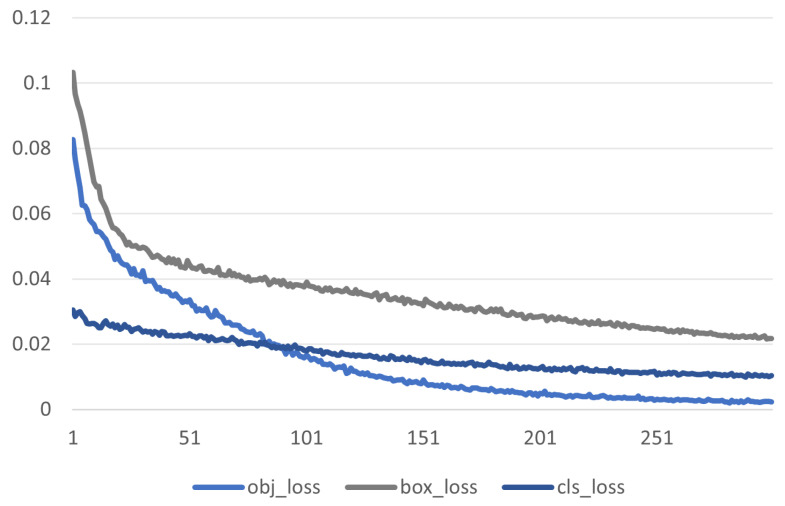
The loss function of Yolo-Pest module training.

**Figure 7:**
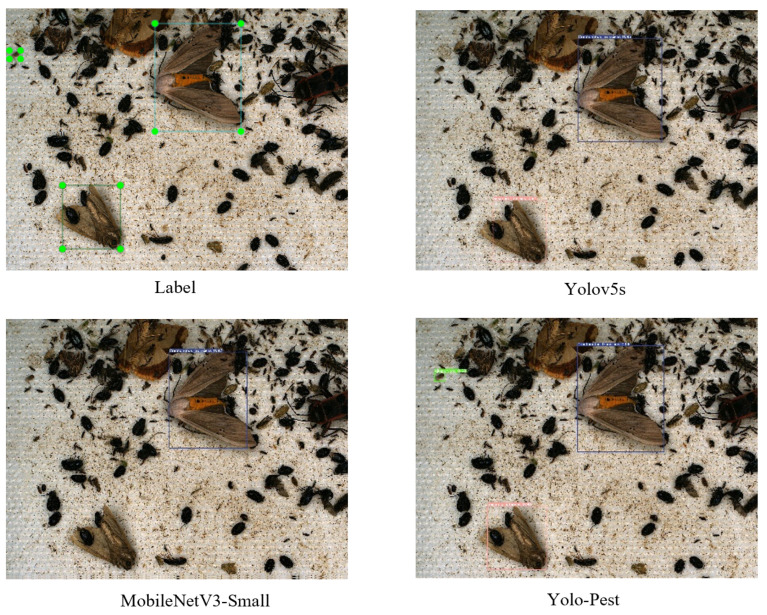
Detection results of the pests (Yolo-Pest found all the pest targets in the green marked box).

Finally, we give a comparison between the Yolo-Pest detectors and the original detectors in Table 5 and Table 6. In Table 5, a large dataset, IP102, is used to verify that the detection accuracy of normal-size targets is not lost in the improvement of small-size targets for Yolo-Pest. The detection frame generally accounts for more than 80% of the images in the IP102 dataset, which is suitable for image classification and belongs to normal- or large-size target datasets in target detection. According to the experimental data, Yolo-Pest maintains relatively high precision after reducing the amount of model data and achieves an excellent balance between the detection accuracy and the model calculation speed for the dataset of large- and normal-size targets. In Table 6, we selected some detectors with similar model complexities and numbers of parameters in the Teddy Cup pest dataset for comparison. The mAP0.5 of Yolo-Pest can reach 91.9% in the Teddy Cup pest dataset, which is nearly 8% higher than that of Yolov5s. The loss function is shown in Figure 6. The recognition accuracy of small target pests is improved, and the recognition accuracy of the normal targets is ensured. This can be visually observed in Figure 7.

## 5. Conclusions

In summary, this paper introduces a simple and easy-to-operate data enhancement method, random background enhancement, which can have a good feature expansion effect on a specific dataset. Compared to the copy-and-paste method, this enhancement focuses on complex backgrounds. They can be used simultaneously on datasets that meet the conditions to achieve a better enhancement effect. In this paper, a novel controllable channel residual feature extraction module, the CAC3 module, is designed. By controlling the number of feature graph channels of the input residuals, the ability of the module to extract features from the input feature graph and the complexity of the model is adjusted. In small target detection, the stacking of residual blocks cannot play its role. By increasing the ratio of residual edges to output channels of the residual structures through the CAC3 module, more features can be retained to improve model accuracy and reduce the number of model parameters and the amount of calculation. We also added the SE attention mechanism and ConvNeXt to combine the features of the ViT model to build Yolo-Pest, an improved YOLO model for pest identification. The mAP0.5 improved by 7.9% in the small-size pest dataset from Teddy Cup, and the identification accuracy was not reduced in the IP102 normal-size pest dataset. The model proposed in this paper is suitable for the detection of mixed-size pest targets on a complex background. Compared with the original Yolov5s model, the accuracy of the detection of small target pests has been improved, and the overall accuracy and robustness of the model have been promoted. Compared with other classical detection models, Yolo-Pest can have a better balance between performance and accuracy. In the case of a similar number of parameters, the model can reach the optimal level.

## Figures and Tables

**Table 1:** Comparison of different backbones in Yolov5s.

Backbone	mAP_0.5_	mAP_0.95_	Param.	Complexity (FLOPs)
CAC3 + DarkNet53 (*e* = 0.25)	86.4%	71.4%	5.9 M	13.0 G
CAC3 + DarkNet53 (*e* = 1.0)	81.2%	72.0%	9.5 M	19.3 G
C3 + DarkNet53	83.7%	71.5%	7.1 M	16.2 G
Hornet [38] + DarkNet53	85.7%	71.2%	9.3 M	21.1 G
Mobilenet [39]	86.1%	70.8%	2.2 M	3.2 G
Regnet [40]	76.1%	59.6%	5.0 M	9.2 G

**Table 2:** Comparison of the performance of different residual feature extraction modules in neck layer.

Moudle	mAP${}_{0.5}$	mAP${}_{0.95}$	Param.	Complexity (FLOPs)
C3 + Bottleneck	83.7%	71.5%	7.1 M	16.2 G
C3 + ConvNext block (ours)	87.7%	71.7%	6.9 M	15.8 G
C3 + Hornet block	85.6%	72.2%	7.3 M	16.7 G
ConvNeXt block	82.8%	70.4%	10.7 M	29.8 G
Hornet block	82.0%	71.1%	9.5 M	19.3 G

**Table 3:** Performance of different attention modules.

Attentions	Param.	P.&R.	mAP${}_{0.5}$	mAP${}_{0.95}$	Complexity (FLOPs)
SE	7.13 M	94.8% 76.8%	85.5%	73.6%	16.2 G
CA	7.12 M	94.7% 75.9%	84.4%	72.0%	16.2 G
CBAM	7.13 M	94.1% 76.2%	83.6%	72.1%	16.2 G
SK [41]	29.2 M	95.0% 76.2%	83.3%	72.3%	33.9 G

**Table 4:** The ablation studies of three implements: CAC3, ConvNeXt block, and attention module.

Models	Param.	mAP_0.5_	mAP_0.95_	Complexity (FLOPs)
Baseline (Yolov5s)	7.1 M	83.7%	71.5%	16.2 G
Baseline + CAC3 (*e* = 0.25)	5.9 M	86.4%	71.4%	13.0G
Baseline + ConvNeXt block	6.9 M	87.7%	71.7%	15.8 G
Baseline + ConvNeXt block + SE	6.9 M	90.4%	75.0%	15.9G
Baseline + CAC3 + ConvNeXtblock + SE (*e* = 0.25)	5.8 M	90.6%	74.6%	12.7 G
Baseline + CAC3 + ConvNeXtblock + SE (*e* = 0.275)	5.9 M	91.9%	72.5%	12.9G
Baseline + CAC3 + ConvNeXtblock + SE (*e* = 0.3)	6.0 M	91.0%	74.3%	13.1G

**Table 5:** Model performance on the IP102 test set.

Detectors	Params (M)	FPS (img/s)	mAP${}_{0.5}$
GhostNet [42]	4.03	1768.49	39.68
ShuffleNetV2	5.55	1686.72	43.63
ResNet18	11.22	1577.33	46.85
MobileNetV3-Large	4.33	1612.18	47.44
DenseNet121 [43]	7.05	684.80	56.1
Yolov5s	7.1	158.70	57.0
**Yolo-Pest (ours)**	5.8	166.67	57.1

**Table 6:** Model performance on the Teddy Cup pest dataset.

Detectors	Params (M)	Complexity (FLOPs)	mAP${}_{0.5}$
Yolov3-tiny	8.7	13.0 G	82.3
Yolov4-mish	9.2	21.0 G	80.4
Yolov5s	7.1	16.2G	83.7
MobileNetV3-Small	2.2	3.2 G	87.9
RepVGG-A0 [44]	8.3	1.4 G	84.1
**Yolo-Pest (ours)**	5.8	12.9 G	91.9

## Data Availability

Not applicable.
